# Courtship Song Does Not Increase the Rate of Adaptation to a Thermally Stressful Environment in a *Drosophila melanogaster* Laboratory Population

**DOI:** 10.1371/journal.pone.0111148

**Published:** 2014-11-03

**Authors:** Larry G. Cabral, Brett Holland

**Affiliations:** Department of Biological Sciences, California State University, Sacramento, California, United States of America; CNRS, France

## Abstract

Courtship song in *D. melanogaster* contributes substantially to male mating success through female selection. We used experimental evolution to test whether this display trait is maintained through adaptive female selection because it indicates heritable male quality for thermal stress tolerance. We used non-displaying, outbred populations of *D. melanogaster* (*nub^1^*) mutants and measured their rate of adaptation to a new, thermally stressful environment, relative to wild-type control populations that retained courtship song. This design retains sexually selected conflict in both treatments. Thermal stress should select across genomes for newly beneficial alleles, increasing the available genetic and phenotypic variation and, therefore, the magnitude of female benefit derived from courtship song. Following introduction to the thermally stressful environment, net reproductive rate decreased 50% over four generations, and then increased 19% over the following 16 generations. There were no differences between the treatments. Possible explanations for these results are discussed.

## Introduction

The relationship between sexual fitness (mating and fertilization success) and population fitness (approximated by net reproductive rate) is not generally understood. Theory indicates that sexual selection can increase beneficial allele fixation [1], deleterious allele removal [1], [2]–[4], and the rate of adaptation to novel environments [5]–[6]. Experiments have been ambiguous. In *D. melanogaster* sexual selection sometimes removes specific deleterious marker mutations (one of one [7], five of eight [8], four of six [9], and zero of six [10]). Attempts to measure sexual selection's effect on non-specific mutational load have been mixed. Bulb mite populations held under relaxed viability and fecundity selection showed no improvement in the presence of sexual selection [11]. With the addition of ionizing radiation, sexual selection increased fitness [12]. But when reintroducing viability and fecundity selection, sexual selection no longer showed a detectable benefit [13]. When natural selection was relaxed in *D. serrata*, sexual selection improved productivity [14]. Dung beetles were exposed to ionizing radiation and then held with/without sexual selection for two generations. Male strength and female productivity were both higher in the sexually selected treatment [15]. Sexual selection did not increase the rate adaptation to a thermally stressful environment in *D. melanogaster* [16], or novel larval food resource in *D. serrata* [17]. Sexual selection did increase the rate of adaptation of a seed beetle to a novel host plant, yet, decreased fitness when maintained on their ancestral host [18].

In a particularly thorough experiment, *D. melanogaster* populations were exposed to EMS, then held with/without sexual selection for 60 generations, at which point the populations were evaluated in both mating environments. Net reproductive rate actually went down in the sexually selected populations, apparently because the costs of sexual selection exceeded any benefits [19]. With sexual selection comes intersexual conflict, which has sometimes favored the evolution of male traits that directly harm females. [20]–[29]. A few experiments with *D. melanogaster* have assessed the direct costs and indirect benefits, finding that the net effect was substantially negative [30]–[32]. The inconsistent results among experiments designed to find benefits to females of sexual selection, may, in part, be due to a lack of control of sexual conflict. Most of those experiments removed sexual selection through enforced monogamy with random mate assignment. Under monogamy, the reproductive success of a mating pair is identical. Therefore, ancestral sources of conflict are new opportunities for adaptation. As sexually antagonistic, female-harm, alleles are removed under monogamy, fitness measures may improve despite the concurrent removal of any benefits of sexual selection [23]. Despite the difference in net reproductive rate, Hollis and Houle [19] found no difference in egg-to-adult viability or fecundity, also illustrating the difficulty of drawing inferences about fitness from its components, where measurement context may differ [19], and individual measures may be less sensitive, or inconsistent.

Within intersexual selection, the 'good genes' hypothesis posits that females select mates with superior non-sexual genetic quality [33], [34], revealed by condition-dependent displays [35]. Those females who happen to prefer such displays produce offspring with superior genomes. Courtship display and preference are both directionally selected. Condition dependence of the display should be an outcome of directional selection on display magnitude through genic capture [2]. A number of empirical studies have found positive correlations between sire attractiveness and offspring fitness components, typically viability [36]–[41]. Interpreting fitness components, such as offspring viability, may be also be problematic due to the influence of male seminal fluid. For example, *T. oceanicus* males vary in their investment in their accessory glands and there is a positive relationship between such investment and the viability of their offspring. One product of their accessory glands, prostaglandin synthetase, stimulates increased female investment per ovule, which increases offspring viability. There is a trade-off between offspring viability and female life-time fecundity. Therefore, male manipulation is apparently moving females away from their fitness optima [42]. In summary, understanding the evolution of male courtship traits may be hampered from a lack of control for coevolutionary conflict in monogamy/polyandry designs, the difficulty of measuring net reproductive rate, and potentially, marginal signal-to-noise.

In male *Drosophila*, individual wing vibrations directed at females (song) is a conspicuous component of courtship and important to mating success [43]–[47]. Wingless males suffer much lower mating success, which is partially rescued by playing artificial song [48]–[50]. Artificial reduction of wing area (environmentally induced, artificial selection, or partial amputation) diminishes song success approximately linearly [46].

Here, we remove courtship song in replicate *D. melanogaster* populations while retaining it in control populations. Both, treatment and control populations are maintained under a sexually competitive environment, where sexual selection and conflict are otherwise fully present. We used an outbred, laboratory adapted population into which the recessive nubbin (*nub^1^*) mutation was introgressed through approximately 20 cycles of backcrossing, making the *nub^1^* population differ from the outbred, wild-type population by less than 1×10^−6^ [51]. This mutation greatly reduces the wing (cell number), deforms the remaining tissue into a folded clump [52], and removes the wing hinge [53] (images available at http://flybase.org/reports/FBal0013178.html). While mating rates are diminished due to *nub^1^* mutation's removal of courtship song, no deficiency in fertility has been observed, due the excess mating that occurs in this species [54].

To increase the opportunity for the good genes process, all populations were exposed to low-grade thermal stress. Conformation determines protein function, membrane fluidity and enzyme catalytic function, and is substantially affected by the elevation of a few degrees Celsius [55]. Most loci in outbred populations possess substantial low frequency variation. Thermal stress should select those alleles that are more thermally-stress tolerant across innumerable loci. Secondary changes in the environment (e.g., humidity, food hydration, flora, etc.) are also potential sources of selection. Natural clines of thermal adaptation occur in this species [56]. The degree of thermal stress used is within the range encountered by *D. melanogaster* in nature and below that which induces heat shock [57]. Courtship song is sensitive to temperature [58] and, therefore, is potentially an indicator of male condition and thermal stress tolerance. If courtship song is an honest signal of heritable male quality, then the wild-type populations should evolve thermal tolerance faster than the nubbin populations because of the additional level of selection on the wild-type males that occurs through wing song evaluation [59]. Adaptation (net reproductive rate) was measured *in situ* as the number of offspring that survive and are available for entry into the next generation. This measure includes female fecundity, offspring survival, and development rate. The experiment was conducted under low-density conditions in which resources were not limiting. All populations were introduced to the control environment (25°C) for five generations, and then transferred to the thermally stressful environment for 21 generations. Net reproductive rate was measured every generation.

## Materials and Methods

### Generation of stocks

The experiments were carried out with a large, outbred population that had been adapting to a controlled laboratory environment for over 400 generations. This wild-type population was established in 1988 from 400 mated females that were collected in central California by L. Harshman. The nubbin population was derived from the wild-type population by Alison Pischedda and Adam Chippindale [51] through approximately 20 cycles of back-crossing of the nubbin into the wild-type population, such that the nubbin locus was within an essentially wild-type genome. This was completed in 1997. Both populations were subsequently maintained at N_e_>5000, at 25°C, on cornmeal/molasses/killed-yeast medium, seeded with live yeast, with a 12 h light: 12 h dark diurnal cycle, and a 14-day generation cycle. The experimental protocol, begun in 2005, maintained these conditions except as noted otherwise below. The nubbin and wild-type populations were generously provided by William R. Rice and Tristin A.F. Long.

### Creating Populations Prior to Thermal Stress

Four samples from both nubbin and wild-type populations were taken to form four replicates from each parent population (n = 105 adults of each sex/replicate). Each vial (95×27.5 mm) contained 7 males and 7 females, 10 ml of medium, and was seeded with live yeast. Adult flies were cultured (day 0) overnight and discarded. The eggs deposited overnight constituted the beginning of the experimental populations. The populations were maintained in this manner, under their ancestral laboratory conditions over the subsequent five generations.

### Standard Culturing Procedure

All adults were counted at least daily as they emerged. Adults emerging on the day of maximum eclosion (day 10, at 25°C) were transferred to a common container, lightly anesthetized (30 seconds of CO_2_), divided into 5 aliquots, and allowed several minutes to recover. Adults were then transferred to food vials (7 flies of each sex/vial, n = 105 adults of each sex), using approximately 4 minutes of CO_2_. After two days (day 12) flies were transferred to fresh, yeasted, food vials. On day 14/0 all flies were transferred to fresh food vials for two hours, producing approximately 100 eggs per vial. After laying eggs the adults were removed, dead females (if any) were counted and the males and females were then discarded. The above cycle was reiterated in subsequent generations.

### Initiating the Thermal Stress Regime

**Figure 1:**
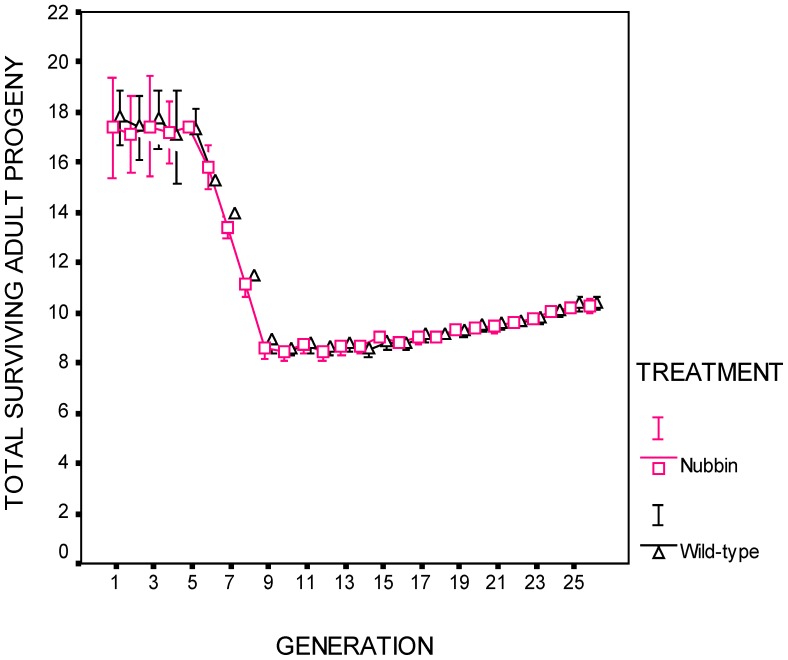
Total surviving adult progeny per female. Populations entered the thermal stress regime in generation 6. There was no difference between treatments (p = 0.64). Error bars are ± one standard error. See Table 1b.

The populations were maintained five generations at their ancestral temperature (25°C). At the beginning of generation 6, eggs from all populations were introduced to a thermally stressful environment. The sensitivity of *D. melanogaster* to thermal stress varies with developmental stage (reviewed in [60]). The thermal regime reflects this variation. Egg deposition by adult females and early embryo development (day 0) occurred at 32°C; larval development and early pupation (days 1–3) occurred at 33°C; later pupal development and early adult stages (days 4–11) occurred at 28°C (males are sterile when developing above 28.5°C); courtship and mating (days 12–13) occurred at 31°C (courtship and mating could also occur on day 0 at 32°C). The thermal protocol reduced female productivity (total number of adult offspring per female) by approximately 50% (Fig. 1).

Development rate in *D. melanogaster* increases with temperature until approximately 28°C, after which it begins to slow due to the rapidly increasing stress [61]. As a result, 80% of thermally stressed progeny emerge on day 9. The adults used for mating were taken from the day 9 collection. All emerging adults were counted. Those emerging before or after the day 9 collection were discarded (8% and 12% of total, respectively). The above protocol was reiterated over subsequent generations. A total of 21 generations of thermal stress selection data was collected. The approximate number of generations was chosen prior to starting the experiment and was based on results of a selection experiment that used the same stress protocol [7].

### Measuring Thermal Adaptation

**Figure 2:**
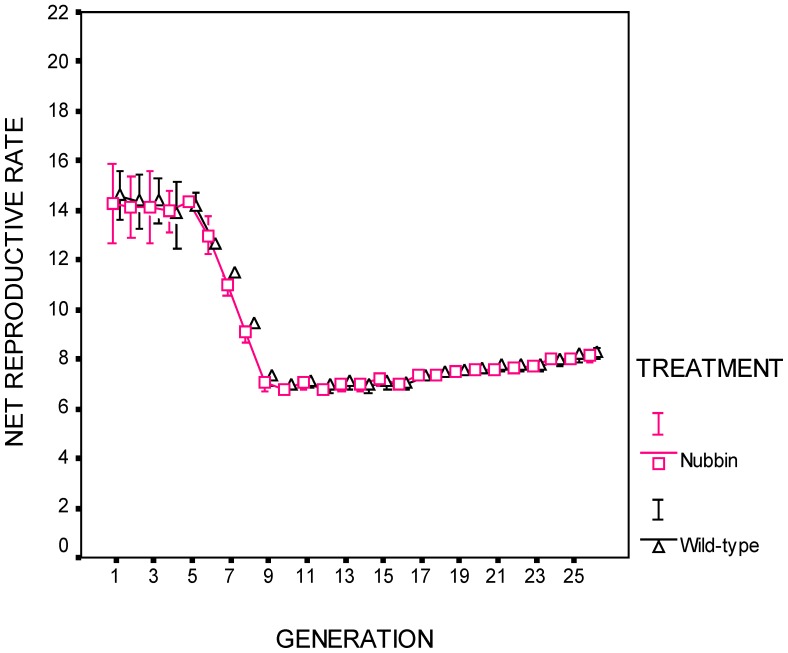
Net reproductive rate per female. This measure includes all adults that were available for collection each generation during the normal collection period. Populations entered the thermal stress regime in generation 6. There was no difference between treatments (p = 0.63). Error bars are ± one standard error. See Table 1a.

Adaptation of the experimental lines was measured *in situ* each generation. Two measures were made: net reproductive rate, consisting of all adult progeny that were available for collection during the normal collection period (those emerging through day 9) (Fig. 2). This measure includes female fecundity and viability and development rate of their offspring. The second measure, productivity, consists of the total number of adult progeny (identical to the previous measure except that it also includes the slow developing offspring emerging after day 9) (Fig. 1).

### Statistical Analysis

Univariate analysis of covariance (ANCOVA) was used to assess statistical significance of thermal adaptation between treatments, using adult progeny as the dependent variable, treatment as a fixed factor, and time (generation number) as the covariate. To avoid pseudoreplication, independent lines (N = 4 per treatment) were used as the data for statistical analysis rather than the individual flies that generated these treatment measures. A normal distribution of the data can be inferred because each measure is an average (or a total) over a large number of contributing individuals. SPSS 13.0 software was used to analyze the data.

## Results

### Thermal Stress

The deleterious effects of thermal stress were observable during generations 6–10. Net reproductive rate and productivity declined to approximately 50% of their starting levels (Figures 1–2).

### Thermal Adaptation

(Generations 10–26) There was no difference between treatments in productivity (Fig. 1; Table 1b), net reproductive rate (Fig. 2; Table 1a), or maternal survival (Table 1c). The covariate, time, was significant for both treatments for productivity (*p*<0.001) and net reproductive rate (*p*<0.001) (Fig. 1, 2; Table 1a, b). For reference, the wild-type treatment populations' net reproductive rate increased by 19% between generations 10 and 26, while productivity increased 21% during the same period. Data is archived at http://www.csus.edu/faculty/h/holland/docs/Puplications/Cabral.Holland.DataArchivePublic.pdf

**Table 1:** Analysis of covariance of treatment and time with respect to net reproductive rate, total surviving adult progeny, and female survival.

DependentVariable		Source	d.f.	Sum of Squares	Mean Square	*F*-value	*P*-value
a) Net Reproductive Rate							
		TIME	1	20.7	20.7	109	<.001
		TREATMENT	1	.044	.044	.229	.633
		Error	141	26.9	.191		
b) Total Surviving Adult Progeny							
		TIME	1	43.7	43.7	163	<.001
		TREATMENT	1	.058	.058	.214	.644
		Error	141	37.9	.269		
c) Maternal Survival							
		TIME	1	.434	.434	.173	.678
		TREATMENT	1	4.00	4.00	1.60	.208
		Error	141	353	2.50		

## Discussion

This is the first study we know to measure the effect of a specific male courtship display on the rate adaptation to a new environment. The estimated adaptation to the new environment indicated by productivity and net reproductive rate is conservative because adaptation from the onset of stress (generation 6) through generations 9–10 was masked by the effects of physiological deterioration within the thermally stressful environment. The adaptation itself demonstrates heritable genetic variation for net reproductive rate and productivity was present. However, the hypothesized benefit of a male courtship ornament was not detected. Four non-mutually exclusive explanations for these results are: the stressor itself interfered with the good genes process; the *nub^1^* population has undergone compensatory evolution; insufficient sensitivity of the experiment; and the absence of the good genes process with respect to courtship song.

Small changes in temperature appear to have pervasive effects on small animal physiology, including sensory systems [62]. The thermal stress of the range used is encountered by wild populations of flies and does not induce heat shock [57]. It does not cause male sterility [16]. Courtship and mating rates of *D. melanogaster* do not appear to be substantially altered within the temperature range used here [63]. One can never know that an environmental parameter, or mutation, has no effect on female ability to discern information about male quality. In general, environmental and genetic stresses will affect both sexes. Therefore, it is an implied aspect of the good genes hypothesis that females will also be able to perform their screening function under the same conditions experienced by males. Given the pervasive occurrence and significance of temperature stress, it would seem, *a priori*, like the sort of environmentally induced stressor that females should be selected to be sensitive to with regard to the good genes process. However, many experiments conducted under a variety of environments must be performed in order to determine the extent and significance of the good genes process.

Compensatory evolution in the nubbin population may have changed female focus to other courtship behaviors (e.g., chasing, tapping, licking, orienting towards females, and copulation attempts) to compensate for the lack of song. The nubbin population used here was studied for such compensatory changes [51]. After 150–180 generations, the nubbin males did significantly adapt. In competition against wild-type males for nubbin females the nubbin males obtained 43% as many matings as wild type males (Fig 2a in [51]). In a newly created nubbin population, males obtained only 29% as many matings as wild-type. In summary, after 150–180 generations, the nubbin mutation still reduces male mating success by 57% relative to otherwise essentially identical wild-type males. Therefore, females remained very sensitive to the presence of male wings and, presumably, courtship song. The experiments reported here occurred approximately contemporaneously with those of Pischedda and Chippindale [51].

The benefit of courtship song may be too small to be detected with this design. Theoretical [64]–[66] and experimental work (reviewed in [39]) indicates that such benefits might be small despite the use of thermal stress. A more long-term experiment may be necessary to detect the benefits of song. There are innumerable traits that may reveal underlying heritable quality. We have removed only one of them. A design that removed multiple traits simultaneously could improve the signal.

Song itself may not currently be maintained through the good genes process. Courtship song selection may be a self-reinforcing (runaway) process in which females select males who will in turn produce sexy sons who are not otherwise better adapted [33]. Courtship song may have been selected through sensory bias [67]–[71]. This model posits that decisions (e.g., whether or not to mate) are the result of innumerable inputs (internal/physiological and those conveyed through external sensors). Sensory systems, like all traits, have incidental qualities that render them vulnerable to exploitation. The exploitation of sensory bias [68]–[70] through song may be sexually antagonistic. There are inevitable costs of copulating: predation and STD exposure [72], seminal fluid components that are toxic [21], such as sex peptide [74], or manipulative, such as Acp26Aa [73] and prostaglandin synthetase [42], [75]. Male displays may simply induce females to mate sub-optimally. This could result in a coevolutionary race in which females are selected for resisting the influence of deleterious displays and males are in turn selected for super-stimulating female sensory biases [76].

An incidental finding may be relevant outside the context of sexual selection. It took four generations of heat stress before net reproductive rate stopped declining. This may be relevant to any experiment that compares populations from different environments. The number of generations needed to eliminate differences in the direct environmental effects on phenotype may have to be determined. This work may also be relevant to studies of the impact of environmental change on populations with slow generation times, where current observations of population attributes might lag the effects of the current environment by several generations.

## References

[1] Whitlock MC (2000) Fixation of new alleles and the extinction of small populations: Drift load, beneficial alleles, and sexual selection. Evolution 54: 1855–1861. 11209765 10.1111/j.0014-3820.2000.tb01232.x

[2] Rowe L, Houle D (1996) The capture of genetic variance by condition dependent traits. Proc. R. Soc. B. 263: 1415–1421.

[3] Agrawal AF (2001) Sexual selection and the maintenance of sexual reproduction. Nature 411: 692–695. 11395771 10.1038/35079590

[4] Whitlock MC, Agrawal AF (2009) Purging the genome with sexual selection: reducing mutation load through selection on males. Evolution 63: 569–582. 19154364 10.1111/j.1558-5646.2008.00558.x

[5] Lorch PD, Proulx S, Rowe L, Day T (2003) Condition-dependent sexual selection can accelerate adaptation. Evol. Ecol. Res. 5: 867–881.

[6] Candolin U, Heuschele J (2008) Is sexual selection beneficial during adaptation to environmental change? Trends Ecol Evol 23: 446–452. 18582989 10.1016/j.tree.2008.04.008

[7] Hollis B, Fierst JL, Houle D (2009) Sexual selection accelerates the elimination of a deleterious mutant in *Drosophila melanogaster* . Evolution 63: 324–333. 19154371 10.1111/j.1558-5646.2008.00551.x

[8] Sharp NP, Agrawal AF (2008) Mating density and the strength of sexual selection against deleterious alleles in *Drosophila melanogaster* . Evolution 62: 857–867. 18221380 10.1111/j.1558-5646.2008.00333.x

[9] Clark SCA, Sharp NP, Rowe L, Agrawal AF (2012) Relative effectiveness of mating success and sperm competition at eliminating deleterious mutations in *Drosophila melanogaster* . PLoS One 7: e37351. 22662148 10.1371/journal.pone.0037351 PMC3360693

[10] Arbuthnott D, Rundle HD (2012) Sexual selection is ineffectual or inhibits the purging of deleterious mutations in *Drosophila melanogaster* . Evolution 66: 2127–37. 22759290 10.1111/j.1558-5646.2012.01584.x

[11] Radwan J, Unrug J, Snigorska K, Gawronska K (2004) Effectiveness of sexual selection in preventing fitness deterioration in bulb mite populations under relaxed natural selection. J. Evol. Biol. 17: 94–99. 10.1046/j.1420-9101.2003.00646.x 15000652

[12] Radwan J (2004) Effectiveness of sexual selection in removing mutations induced with ionizing radiation. Ecol. Lett. 7: 1149–1154.

[13] Plesnar A, Konior M, Radwan J (2011) The role of sexual selection in purging the genome of induced mutations in the bulb mite (Rizoglyphus robini) Evolutionary Ecology Research. 13: 209–216..

[14] McGuigan K, Petfield D, Blows MW (2011) Reducing mutation load through sexual selection on males. Evolution 65: 2816–2829. 21967424 10.1111/j.1558-5646.2011.01346.x

[15] Almbro M, Simmons LW (2014) Sexual selection can remove an experimentally induced mutation load. Evolution 68: 295–300.. 24372608 10.1111/evo.12238

[16] Holland B (2002) Sexual selection fails to promote adaptation to a new environment. Evolution 56: 721–730. 12038530 10.1111/j.0014-3820.2002.tb01383.x

[17] Rundle HD, Chenoweth SF, Blows MW (2006) The roles of natural and sexual selection during adaptation to a novel environment. Evolution 60: 2218–2225. 17236415

[18] Fricke C Arnqvist G (2007) Rapid adaptation to a novel host in a seed beetle (*Callosobruchus maculatus*): the role of sexual selection. Evolution 61: 440–454. 17348953 10.1111/j.1558-5646.2007.00038.x

[19] Hollis B, Houle D (2011) Populations with elevated mutation load do not benefit from the operation of sexual selection. J. Evol. Biol. 24: 1918–1926. 10.1111/j.1420-9101.2011.02323.x PMC3156275 21658188

[20] Parker GA (1979) Sexual selection and sexual conflict InBlumMSandBlumNAeditors Sexual selection and reproductive competition in insectsNew York:Academic Presspp.123–166.

[21] Chapman T, Liddle LF, Kalb JM, Wolfner MF, Partridge L (1995) Cost of mating in *Drosophila melanogaster* females is mediated by male accessory gland products. Nature 373: 241–244. 7816137 10.1038/373241a0

[22] Rice WR (1996) Sexually antagonistic male adaptation triggered by experimental arrest of female evolution. Nature 361: 232–234. 10.1038/381232a0 8622764

[23] Holland B, Rice WR (1999) Experimental removal of sexual selection reverses intersexual antagonistic coevolution and removes a reproductive load. Proc. Natl. Acad. Sci. U. S. A. 96: 5083–5088.. 10.1073/pnas.96.9.5083 PMC21820 10220422

[24] Lung O, Tram U, Finnerty CM, Eipper-Mains MA, Kalb JM, et al (2002) The *Drosophila melanogaster* seminal fluid protein Acp62F is a protease inhibitor that is toxic upon ectopic expression. Genetics 160: 211–224. 11805057 10.1093/genetics/160.1.211 PMC1461949

[25] Martin OY, Hosken DJ (2003) Costs and benefits of evolving under experimentally enforced polyandry or monogamy. Evolution Int J Org Evolution 57: 2765–2772. 10.1111/j.0014-3820.2003.tb01518.x 14761055

[26] Wigby S, Chapman T (2004) Female resistance to male harm evolves in response to manipulation of sexual conflict. Evolution 58: 1028–1037. 15212383 10.1111/j.0014-3820.2004.tb00436.x

[27] Arnqvist G, Rowe L (2005) Sexual conflict. Princeton: Princeton University Press. 325 p.

[28] Friberg U, Lew TA, Byrne PG, Rice WR (2005) Assessing the potential for an ongoing arms race within and between the sexes: Selection and heritable variation. Evolution 59: 1540–1551. 16153039

[29] Lew TA, Rice WR (2005) Natural selection favours harmful male Drosophila melanogaster that reduce the survival of females. Evol. Ecol. Res. 7: 633–641.

[30] Stewart AD, Morrow EH, Rice WR (2005) Assessing putative interlocus sexual conflict in Drosophila melanogaster using experimental evolution. Proc. R. Soc. B 272: 2029–2035. 10.1098/rspb.2005.3182 PMC1559894 16191613

[31] Rice WR, Stewart AD, Morrow EH, Linder JE, Orteiza N, et al (2006) Assessing sexual conflict in the *Drosophila melanogaster* laboratory model system. Philos. Trans. R. Soc. Lond. B Biol. Sci. 361: 287–299. 10.1098/rstb.2005.1787 PMC1569610 16612888

[32] Stewart AD, Hannes AM, Mirzatuny A, Rice WR (2008) Sexual conflict is not counterbalanced by good genes in the laboratory *Drosophila melanogaster* model system. J Evol Biol 21: 1808–1813. 18681915 10.1111/j.1420-9101.2008.01593.x

[33] Fisher RA (1952) The genetical theory of natural selection. New York: Dover Publications.

[34] Williams GC (1966) Adaptation and Natural Selection. Princeton NJ: Princeton University Press.

[35] Zahavi A (1975) Mate selection-a selection for a handicap. J. Theor. Biol. 53: 205–214. 10.1016/0022-5193(75)90111-3 1195756

[36] Partridge L (1980) Mate choice increases a component of offspring fitness in fruit flies. Science 283290–291.

[37] Petrie M (1994) Improved growth and survival of offspring of peacocks with more elaborate trains. Nature 371: 598–599.

[38] Welch AM, Semlitsch RD, Gerhardt HC (1998) Call duration as an indicator of genetic quality in male gray tree frogs. Science 280: 1928–1930. 9632389 10.1126/science.280.5371.1928

[39] Møller AP, Alatalo R (1999) Good-genes effects in sexual selection. Proc. R. Soc. Lond. B 266: 85–91.

[40] Forsman A. Hagman M (2006) Calling is an honest indicator of paternal genetic quality in poison frogs. Evolution 60: 2148–2157. 17133871

[41] Firman RC, Simmons LW (2012) Male house mice evolving with post-copulatory sexual selection sire embryos with increased viability. Ecol. Lett. 15: 42–46. 10.1111/j.1461-0248.2011.01706.x 22011211

[42] Simmons LW, García-González F (2008) Evolutionary reduction in testes size and competitive fertilization success in response to the experimental removal of sexual selection in dung beetles. Evolution 62: 2580–2591. 18691259 10.1111/j.1558-5646.2008.00479.x

[43] Sturtevant AH (1915) Experiments on sex recognition and the problem of sexual selection in *Drosophila*. J. Anim. Behav. 5: 351–366.

[44] Spieth HT (1952) Mating behavior within the genus *Drosophila* (Diptera). Bulletin of the American Museum of Natural History 99: 395–474.

[45] Bastock M, Manning A (1955) The courtship of Drosophila melanogaster. Behaviour 5: 85–111.

[46] Ewing AW (1964) The influence of wing area on the courtship behavior of Drosophila melanogaster. Anim. Behav. 12: 316–320.

[47] Hall JC (1994) The mating of a fly. Science 264: 1702–1714. 8209251 10.1126/science.8209251

[48] Bennet-Clark HC, Ewing AW (1969) Pulse interval as a critical parameter in the courtship song of Drosophila melanogaster. Animal Behaviour 17: 755–759.

[49] von Schilcher F (1977) A mutation which changes courtship song in Drosophila melanogaster. Behavior Genetics 7: 251–259. 405965 10.1007/BF01066278

[50] Talyn B, Dowse H (2003) FORTRAN program generates effective artificial courtship song. Drosophila Information Service. 86: 148–149.

[51] Pischedda A, Chippindale A (2005) Sex, mutation and fitness: asymmetric costs and routes to recovery through compensatory evolution. J Evol Biol 18: 1115–1122. 16033585 10.1111/j.1420-9101.2005.00915.x

[52] Ng M, Diaz-Benjumea FJ, Cohen SM (1995) nubbin encodes a POU-domain protein required for proximal-distal patterning in the Drosophila wing. Development 121: 589–599. 7768195 10.1242/dev.121.2.589

[53] Perea D, Molohon K, Edwards K, Díaz-Benjumea FJ (2013) Multiple roles of the gene zinc finger homeodomain-2 in the development of the Drosophila wing. Mechanisms of Development 130: 467–481. 23811114 10.1016/j.mod.2013.06.002

[54] Fowler K, Partridge L (1989) A cost of mating in female fruitflies. Nature 338: 760–761.

[55] White FN, Somero G (1982) Temperature and the internal milieu. Physiol. Rev. 62: 41–90. 10.1152/physrev.1982.62.1.40 7034010

[56] James AC, Partridge L (1995) Thermal evolution of rate of larval development in *Drosophila melanogaster* in laboratory and field populations. J. Evol. Biol. 8: 315–330.

[57] Feder ME, Blair N, Figueras H (1997) Natural thermal stress and heat-shock protein expression in *Drosophila* larvae and pupae. Funct. Ecol. 11: 90–100.

[58] Shorey HH (1962) Nature of the sound produced by *Drosophila melanogaster* during courtship. Science 137677–8. 10.1126/science.137.3531.677 17770950

[59] Proulx SR (1999) Matings systems and the evolution of niche breadth. Am. Nat. 154: 89–98. 10.1086/303218 29587498

[60] Ashburner M (1989) Drosophila a laboratory handbook. Cold Spring Harbor, New York. Cold Spring Harbor Laboratory Press. 1331 p.

[61] David JR, Allemand R, Herrwege V, Cohet Y (1983) Ecophysiology: abiotic factors. In The genetics and biology of Drosophila Carson HL, Ashburner MJ, Thompson JN, editors Vol. 3. . Academic Press. pp 105–170.

[62] Cook B, Hardy RW, McConnaughey WB, Zuker CS (2008) Preserving cell shape under environmental stress. Nature 452: 361–364. 18297055 10.1038/nature06603 PMC2387185

[63] Patton ZJ, Krebs RA (2001) The Effect of Thermal Stress on the Mating Behavior of Three Drosophila Species. Physiological and Biochemical Zoology 74: 783–788. 11731970 10.1086/323327

[64] Kirkpatrick M, Barton NH (1997) The strength of indirect selection on female mating preferences. Proc. Natl. Acad. Sci. USA 94: 1282–1286. 10.1073/pnas.94.4.1282 PMC19782 9037044

[65] Cameron E, Day T, Rowe L (2003) Sexual conflict and indirect benefits. J. Evol. Biol. 16: 1055–1060. 10.1046/j.1420-9101.2003.00584.x 14635921

[66] Chapman T, Arnqvist G, Bangham J, Rowe L (2003) Sexual conflict. Trends Ecol. Evol. 18: 41–47.

[67] West-Eberhard M (1984) Sexual selection, competitive communication and species-specific signals in insects. In T Lewis, ed. Insect communication. Toronto: Academic Press. pp. 283–324.

[68] Ryan MJ, Fox JH, Wilczynski W, Rand AS (1990) Sexual selection for sensory exploitation in the frog Physalaemus pustulosus. Nature 343: 66–67. 2296291 10.1038/343066a0

[69] Ryan MJ (1990) Sexual selection, sensory systems, and sensory exploitation. Oxf. Surv. Evol. Biol. 7: 156–195.

[70] Ryan MJ, Rand AS (1990) The sensory basis of sexual selection for complex calls in the tungara frog, *Physalaemus pustulosus* (sexual selection for sensory exploitation). Evolution 44: 305–14. 28564368 10.1111/j.1558-5646.1990.tb05200.x

[71] Basolo AL (1990) Female preference predates the evolution of the sword in swordtail fish. Science 250: 808–810. 17759973 10.1126/science.250.4982.808

[72] Daly M (1978) The cost of mating. Am. Nat. 112: 771–774.

[73] Herndon LA, Wolfner MF (1995) A *Drosophila* seminal fluid protein, Acp26Aa, stimulates egg laying in females for 1 day after mating. Proc. Natl. Acad. Sci. USA 92: 10114–10118. 10.1073/pnas.92.22.10114 PMC40746 7479736

[74] Wigby S, Chapman T (2005) Sex peptide causes mating costs in female Drosophila melanogaster. Curr Biol 15: 316–321. 15723791 10.1016/j.cub.2005.01.051

[75] Sakaluk SK (2006) Cryptic sexual conflict in gift-giving insects: Chasing the chase-away. American Naturalist 167: 94–104. 10.1086/498279 16475102

[76] Holland B, Rice WR (1998) Chase-away sexual selection: antagonistic seduction versus resistance. Evolution 52: 1–7. 28568154 10.1111/j.1558-5646.1998.tb05132.x

